# The aims and effectiveness of communities of practice in healthcare: A systematic review

**DOI:** 10.1371/journal.pone.0292343

**Published:** 2023-10-10

**Authors:** Alexander P. Noar, Hannah E. Jeffery, Hariharan Subbiah Ponniah, Usman Jaffer

**Affiliations:** 1 Department of Surgery and Cancer, Faculty of Medicine, Imperial College London, London, United Kingdom; 2 Highgate Mental Health Centre, Camden and Islington NHS Foundation Trust, London, United Kingdom; 3 Department of General Surgery, East and North Hertfordshire NHS Trust, Stevenage, United Kingdom; 4 Department of Vascular Surgery, Imperial College Healthcare NHS Trust, London, United Kingdom; Aichi Prefectural Mikawa Aoitori Medical and Rehabilitation Center for Developmental Disabilities, JAPAN

## Abstract

Communities of practice (CoPs) are defined as "groups of people who share a concern, a set of problems, or a passion about a topic, and who deepen their knowledge and expertise by interacting on an ongoing basis". They are an effective form of knowledge management that have been successfully used in the business sector and increasingly so in healthcare. In May 2023 the electronic databases MEDLINE and EMBASE were systematically searched for primary research studies on CoPs published between 1st January 1950 and 31st December 2022. PRISMA guidelines were followed. The following search terms were used: community/communities of practice AND (healthcare OR medicine OR patient/s). The database search picked up 2009 studies for screening. Of these, 50 papers met the inclusion criteria. The most common aim of CoPs was to directly improve a clinical outcome, with 19 studies aiming to achieve this. In terms of outcomes, qualitative outcomes were the most common measure used in 21 studies. Only 11 of the studies with a quantitative element had the appropriate statistical methodology to report significance. Of the 9 studies that showed a statistically significant effect, 5 showed improvements in hospital-based provision of services such as discharge planning or rehabilitation services. 2 of the studies showed improvements in primary-care, such as management of hepatitis C, and 2 studies showed improvements in direct clinical outcomes, such as central line infections. CoPs in healthcare are aimed at improving clinical outcomes and have been shown to be effective. There is still progress to be made and a need for further studies with more rigorous methodologies, such as RCTs, to provide further support of the causality of CoPs on outcomes.

## Introduction

Medical knowledge is estimated to double every 73 days [1], leaving both physicians and patients with a seemingly insurmountable amount of information to stay on top of. This essentially means those involved in healthcare have to become skilled at knowledge management, defined as ‘the collection of methods related to creating, sharing, using, and managing the knowledge and information of an organisation’ [2].

One knowledge management strategy that has received significant attention is the theory of communities of practice (CoPs). CoPs are defined as "groups of people who share a concern, a set of problems, or a passion about a topic, and who deepen their knowledge and expertise by interacting on an ongoing basis" [3]. CoPs have a domain of interest, a community of individuals who all share that interest, and a practice consisting of the shared knowledge and skills built up by the community.

Initially described in the business sector, they have been particularly effective as a mechanism for the sharing of tacit knowledge [4]. First described by Polanyi, the Hungarian-British philosopher in 1966 [5], tacit knowledge, in comparison to explicit knowledge, is very difficult to directly codify and share in guidelines. It is best communicated through direct observation and imitation as well as through conversations, stories, and metaphors. The medical profession is a clear example of one where tacit knowledge is constantly used, exemplified by the ‘mindlines’ (rather than guidelines) that practitioners tend to follow [6].

There has been an evolution of the concept, when initially described by Wenger and Lave, they were highly location specific, to a certain office or workspace, where individuals working together would interact, bouncing ideas off each other and helping newer members become fully integrated into the working environment. Over time, the description altered to include those who were not working together in the same physical place, but still shared the same domain of interest and were working on the same set of problems. This opened up the opportunity for virtual CoPs (vCoPs) to be included in the definition, where communities from all over the world interact digitally, producing the same tacit sharing effects as those working in the same physical space.

This review looks to elucidate the aims and effectiveness of CoPs in healthcare as well as communication methods used in these CoPs. We will also show what barriers and facilitators CoPs find when they are implemented in healthcare settings.

## Material and methods

In May 2023 the electronic databases MEDLINE and EMBASE were systematically searched for primary research studies on CoPs published between 1st January 1950 and 31^st^ December 2022. PRISMA guidelines were followed.

The following search terms were used: community/communities of practice AND (healthcare OR medicine OR patient/s). The search was limited to research on human subjects and papers published in the English language. There was no restriction on geographical location.

This review was limited to original research with a focus on CoPs in the healthcare sector. Only papers published in peer- reviewed journals were included. Exclusion criteria were as follows:

- Studies reporting on CoPs in sectors other than healthcare.
- Studies reporting on medical education.
- Studies reporting on multiple interventions
- Case studies.
- Records with no abstracts.
- Study protocols
- Review articles
- News-style or opinion articles, theses and dissertations, and abstracts of conference proceedings without full peer-reviewed papers.

**Fig 1:**
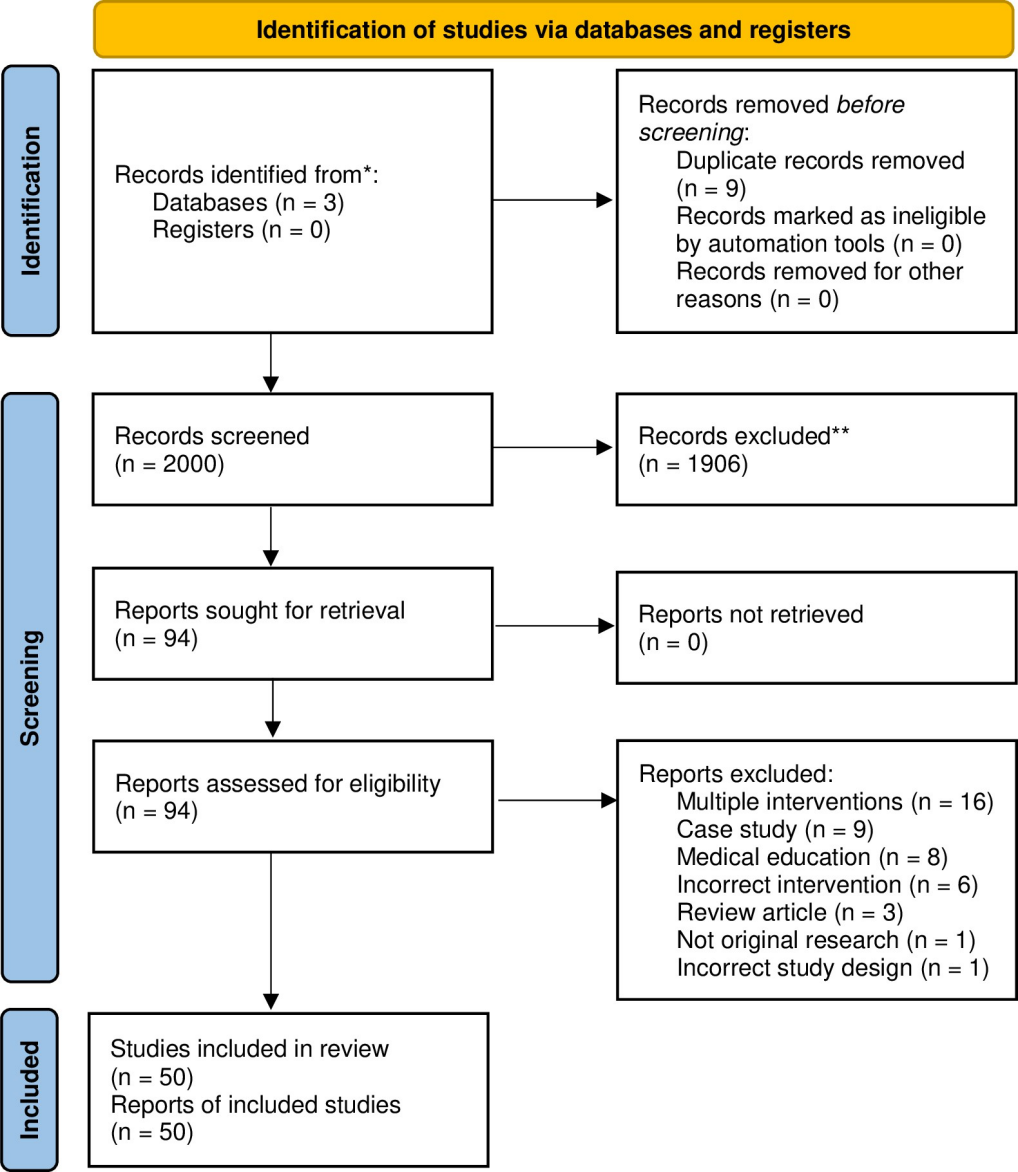
PRISMA flow diagram.

The search was completed using Ovid, and the reference list was uploaded to Covidence. Two authors (APN and HSP) independently reviewed all titles and abstracts, checking against inclusion and exclusion criteria. Relevant papers were marked for retrieval of full text and detailed review. When decisions differed, a final decision was made after discussion between the two reviewers. One author (APN) reviewed and extracted using a standardised template. Reference lists of included studies were also screened. When relevance of the paper was uncertain, or the findings were difficult to extract, APN discussed the paper with UJ. PRISMA flow diagram can be seen in Fig 1.

The following data were extracted: study details (author name, year of publication, country, sample size, study design, study type, data collection method, data analysis method, outcomes measured, barriers/facilitators, and limitations) and description of the CoP (including population, why it was established, how it was established, method of communication, and content shared).

Bias was assessed using the Critical Appraisal Skills Programme (CASP) checklist. Microsoft Excel was used to build tables of the studies included in this review. This review was not registered and a protocol was not prepared. Template data collection forms and data extracted from included studies is available upon request.

## Results

### Results

The database search picked up 2009 studies for screening, of which 94 studies were eligible for full-text review. Of these 50 papers met the inclusion criteria for this systematic review. The most frequent reason for exclusion at this stage was that the study included multiple interventions of which only one was a CoP. Total participants in CoPs across the studies were 12,400, with an average of 282 participants per study (6 studies did not report participant number).

### Country and year of publication

The most common frequent country that the studies were published in was Canada with 12 studies [7, 16, 19, 35, 38, 40, 41, 43, 45, 47, 54, 56], followed closely by the USA with 10 studies [9, 10, 14, 17, 23–25, 48, 51, 55], and the UK with 8 studies [8, 12, 13, 15, 18, 22, 31, 37]. Other notable contributions came from Australia with 6 studies [26, 29, 33, 34, 44, 50] and Spain with 4 studies [20, 27, 39, 42]. All other countries had 2 or less studies. As for year of publication, there was an overall trend of an increasing number of publications in more recent years. 2021 and 2015 had the largest number of studies with 7. 2019, 2018, 2016, and 2014 all have 4 studies. Only 2013, 2005, and 2007 had no studies published in those years.

### The aims of the CoPs

There were a number of themes that emerged from the aims of the CoPs examined in this study (Table 1). The most common by far was to directly improve a clinical outcome, with 19 studies aiming to achieve this. This included disease related factors such as reducing central line infections [9], improving glucose control in critically ill patients [40], and increasing viral suppression rates in HIV [51]. This theme also included many aspects of improving clinical services and workflows such as improving rehabilitation for patients with AF [49], improve pain practices for spinal cord injury patients [43], and improve the falls prevention care for care-home residents [34].

**Table 1:** Aims and effectiveness of included studies.

Ref	Bias	Year of Publication	Authors	Location	Participants	Study Design	Outcome Measure	Aim	Effectiveness
[7]	High	2003	Gagliardi et al.	Canada	22	Non-randomised experimental study	Mixed	To facilitate interaction between community-based general surgeons and oncologists in a tertiary care setting through interactive multidisciplinary rounds.	Feasible to engage remote surgeons in multidisciplinary oncology rounds by videoconference. 25% of participants said that their practice would change.
[8]	Med	2004	Russel et al.	UK	2800	Qualitative Research	Qualitative	To promote evidence based healthcare by linking practitioners with researchers	Communities of practice emerged from the informal email network. The network helped to bridge the gap between research and practice providing the opportunity to collaborate across boundaries.
[9]	Med	2006	Render et al.	USA	/	Non-randomised experimental study	Mixed	To reduce the number of central line infections in hospitals	All sites reduced central line infections by 50% (1.7 to 0.4/1000 line days, p<0.05). Adherence to evidence based practices increased from 30% to nearly 95%.
[10]	Low	2008	White et al.	USA	74	Qualitative Research	Qualitative	To enhance quality of care and safe practices in acute and community care departments in a rural hospital	CoPs enhanced interprofessional practice through improving communications, such as introducing joint care meetings, or information transfer, such as streamlining discharge processes.
[11]	Med	2008	Falkman et al.	Sweden	24	Qualitative Research	Mixed	To improve the ability of oral medicine to share cases and learn from each other due to their geographically dispersed speciality.	The introduction of SOMWeb improved the structure of meetings and their discussions, and a tenfold increase in the number of participants. The platform has been adopted as the national website for continuing education in oral medicine.
[12]	Low	2008	Tolson et al.	UK	24	Non-randomised experimental study	Mixed	To promote evidence-based practice in NHS sites	80% of patient related criteria and 35% of the facilities criteria were achieved. The Revised Nursing Work Index indicated the nurses experienced greater autonomy (p = 0.019) and increased organisational support (p = 0.037).
[13]	Low	2009	Griffiths et al.	UK	19	Qualitative Research	Qualitative	To satisfy the workplace demands that the nurses faced on medical assessment units	The main themes identified regarding the nurses role were organising the clinical space, having professional knowledge, and having the ability to work under pressure.
[14]	Med	2010	Arora et al.	USA	/	Qualitative Research	Mixed	To develop knowledge and skills in provincial primary care providers regarding management of hepatitis C virus	Clinicians report increased competence in all nine abilities for HCV management after 12 months of participation e.g. ability to treat patients with HCV and manage side effects Likert scale average 2.0 to 5.2 (p<0.0001). 98% of respondents thought that ECHO participation had either a moderate or major benefit on enhancing knowledge about management and treatment of patients with HCV. Clinical providers found the case-based learning the most essential source of learning.
[15]	High	2010	Skirton et al.	UK	156	Qualitative Research	Qualitative	To develop standards and a code of practice for genetic counselling to guide professionals in Europe.	The members of the CoP developed a set of professional standards and a code of practice. Suggestions included making genetic counsellor a protected title requiring a master level degree in genetic counselling.
[16]	Med	2011	Burgess et al.	Canada	11	Qualitative Research	Qualitative	To engage nurse practitioners in social investigation, education and actions, and to explore how collaboration advances their role in primary healthcare	CoP helped NPs to build collaborative relationships, enhance practice learning and competence, extend and apply new knowledge, enrich professional identities, and shape health organisational policy and politics. CoP is seen as a major factor for the 100% retention rate of NPs. CoP facilitated exchange of ideas that led to many successful abstract submissions. Participation in the CoP helped build a better sense of the unique identity of being a NP.
[17]	High	2011	Massett et al.	USA	/	Non-randomised experimental study	Quantitative	To help with the issues of oncology clinical trial accrual	AccrualNet has had more than 45000 views, with the Tools and Resources, Conversations, and Training sections being the most viewed. Total content has increased by 69%. Total conversations were 29 with 43 posts.
[18]	Med	2013	Adams et al.	UK	44	Qualitative Research	Qualitative	To facilitate informal learning amoung nurses in community services	The higher performing service (service B) had more time for catch ups in comparison to the lower performing service (service A). An erosion of workplace relationships left them feeling alone and unsupported in service A. Service B phoned around so many nurses went to lunch at the same time. The ideas discussed during catch ups helped staff develop a better understanding of approaches to patient care.
[19]	High	2014	Fung-Kee-Fung et al.	Canada	230	Non-randomised experimental study	Quantitative	To improve cancer care in a regional quality improvement collaborative.	The CoP aided development of a collaboration between hospitals that saw compliance with guidelines improve by 20%, as well as the standardisation of peri-operative pathways in a number of disease sites. Increases in the use of sentinel lymph node biopsy in breast cancer surgery and decreased positive surgical margin rates in prostate cancer were also seen.
[20]	Med	2014	Diaz-Chao et al.	Spain	169	Non-randomised experimental study	Quantitative	To improve primary care and reduce hospital referrals.	Use of the platform improved primary care (p<0.001) and led to fewer hospital referrals (p<0.05). When healthcare staff used social networks and ICT technologies professionally, and had more contact hours with patients, the more the platform was used for communication between primary and hospital care professionals.
[21]	Med	2014	Bindels et al.	Netherlands	13	Qualitative Research	Qualitative	To evaluate the implementation of programs that provide care for frail older people	CoP members had unanticipated concerns regarding the pro-active approach of the programs and older people not being open to receiving care. CoP is a useful strategy as part of an evaluation aimed at improving program implementation. CoP allowed for moral issues of providing care, such as care avoidance, to be discussed, for which there are no guides of how to manage. CoP created a social infrastructure, which allowed for more collaboration.
[22]	Med	2014	Carolan et al.	UK	43	Qualitative Research	Qualitative	To help parents of children and young people with CKD engage in an online platform to aid shared responsibility for condition management.	Evolving communities of child-healthcare practice were identified comprising three components: Parents making sense of clinical tasks, parents executing tasks according to their individual skills, and parents defining task and group members’ worth and creating a personal identity within the community.
[23]	Med	2015	Meins et al.	USA	58	Non-randomised experimental study	Mixed	To provide specialist pain management consultation to community healthcare providers without access to these services locally.	Telepain was determined to be a CoP by displaying the 14 indicators of a CoP described by Wenger. Telepain also enhanced the knowledge of community healthcare provider’s regarding pain management strategies (average score 3.94/4) as well as increasing their confidence (3.77/4).
[24]	Low	2015	Shaikh et al.	USA	31	Qualitative Research	Qualitative	To increase assessment and counseling for childhood obesity prevention	The main challenges to the quality improvement project HEALTH COP were getting staff buy-in, changing ingrained clinical practices, and motivating patients and families. Facilitators were top down requirements for QI, linkages to QI resources, involvement of clinical champions, alignment with existing practices, incorporating a learning system connecting similar clinics, and clear communication channels.
[25]	Med	2015	Heidenreich et al.	USA	305	Randomised Controlled Trial	Quantitative	To aid the enrolment and adoption of the Hospital to Home quality improvement initiative to improve the transition of care for hospitalised patients with heart disease.	54% of hospitals randomised to the CoP intervention arm enrolled patients into Hospital to Home (H2H), compared to 10% in the control arm (p<0.001). Intervention hospitals had more ongoing or planned projects related to H2H (p<0.001). Total cost of CoP facilitation was estimated at $10,200.
[26]	Med	2015	Jefford et al.	Australia	/	Qualitative Research	Qualitative	To trial novel models of post treatment care in cancer patients	Cancer patients found the interventions to be acceptable, appropriate, and effective.
[27]	Low	2015	Lacaster Tintorer et al.	Spain	166	Qualitative Research	Mixed	To improve the communication between primary care and specialist healthcare professionals.	The most important factor for engagement with the CoP was the perceived usefulness for reducing costs of clinical practice. Both perceived usefulness for improving the quality of clinical practice and habitual social media use also helped to drive engagement.
[28]	Med	2015	Dong et al.	International	500	Qualitative Research	Mixed	To aid hand surgeons with continuing professional development	Number of members grew from 38 to 4106. Members perceived the LinkedIn community as user-friendly and easy to use. 42% answered strongly agree, and 37% agree to the question ’How would you rate the overall ease of using the platform?’. System usability scale score 84.6.
[29]	Med	2016	Gullick et al.	Australia	25	Qualitative Research	Qualitative	To build research skills for nurses in busy clinical environments.	The CoP created enduring research relationships and participants described significant value to the research culture that was developed. Many examples of research dissemination and enrolment in doctoral programmes came from participation in the CoP.
[30]	Low	2016	McCreesh et al.	Ireland	12	Qualitative Research	Qualitative	To help physiotherapists working in primary care manage shoulder pain	A desire for peer supports was the strongest motivator for joining. Barriers including not having enough time to engage fully due to work pressures. The access to meetings, the provision of preparation work, and deadlines for the journal clubs were reported as facilitators. Benefits included reported positive clinical practice changes as well as personal growth and development particularly with evidence-based practice skills.
[31]	Low	2016	Wallis et al.	UK	26	Qualitative Research	Qualitative	To improve the management of TB	Participants described the development of a community of practice. The audit promoted local and regional team working, exchange of good practices, and local initiatives to improve care.
[32]	Med	2016	Becerril-Montekio et al.	Mexico	200	Qualitative Research	Qualitative	To strengthen healthcare professionals capacities to acquire, analyse, adapt, and apply research results.	Quality of healthcare was seen as the most important problem of the state departmental health system that represents an obstacle to reach the expected results of maternal health programs. Quality of healthcare and excess of patient demand were seen as the most feasible problems to solve.
[33]	Low	2016	Terp et al.	Australia	11	Qualitative Research	Qualitative	To co-design a smartphone application for use in early schizophrenia care.	The major categories supporting an engaging environment were: a pre-narrative about a community of practice; the room for design is a community of practice; and the community of practice as a practice of special qualities. Participatory design can support and inspire participation and engagement in the development of mental health care with young adults with schizophrenia.
[34]	High	2017	Francis-Coad et al.	Australia	20	Qualitative Research	Mixed	To help reduce the number of falls in residential aged care sites.	The audit conducted by the CoP revealed gaps in practice such as the number low number of residents receiving Vitamin D, the lack of a mandatory falls prevention education for staff, and no falls prevention policy. Actions included requesting that GPs prescribe vitamin D, defining falls, and writing a falls prevention policy.
[35]	Med	2017	Camden et al.	Canada	41	Non-randomised experimental study	Mixed	To improve physical therapists’ self-perceived practice	Self-perceived knowledge, skills, and practice change scores were significantly higher (+0.47, +1.23, and +2.61 respectively; p<0.001) at the end of the CoP compared with the beginning. CoP also significantly impacted belief about capabilities and social influence (+6.64 p<0.002, +5.08 p<0.03 respectively).
[36]	Med	2018	Cheng et al.	International	688	Qualitative Research	Mixed	To encourage collaborative, multi-centre simulation-based research.	The network successfully completed and published numerous collaborative research projects in simulation. INSPIRE has won grant funding for infrastructure support. All 14 of Wenger’s indicators for the presence of a community of practice were found.
[37]	Low	2018	Weiringa et al.	UK	/	Qualitative Research	Qualitative	To allow physicians to discuss patient care and share experiences	Very few posts in the virtual communities of practice referred to explicit guidelines. Instead individual cases highlighted outliers. Tacit, rather than explicit, knowledge was expressed as well as pragmatic reasoning focusing on particular cases. Discussion were reinforced through stories, jokes, and imagery.
[38]	High	2018	Fingrut et al.	Canada	148	Non-randomised experimental study	Quantitative	To decrease barriers to access, foster collaboration, and improve knowledge of guidelines in cancer care.	Participants mostly agreed or strongly agreed that the CoP reduced barriers (76.0%), improved access (82.4%), fostered teamwork (84.5%), improved knowledge (93.3%), improved standards of practice (92.3%), and increased satisfaction in caring for patients (82.9%). The CoP also brought members of the government and hospital administration together with frontline clinicians.
[39]	Low	2018	Lacaster Tintorer et al.	Spain	29	Qualitative Research	Qualitative	To facilitate the communication between primary care and specialist healthcare professionals.	Participants reported that the tool should be integrated into habitual clinical workstations to be of most effect. They also thought contact with specialists should be virtual and that they should be provided with specific time to access the tool.
[40]	Med	2019	Dodek et al.	Canada	272	Non-randomised experimental study	Quantitative	To improve glucose control in critically ill patients	No significant changes to the average hyperglycaemic index, hypoglycaemic events, or standardised mortality rate in response to interventions.
[41]	Med	2019	Glicksman et al.	Canada	275	Non-randomised experimental study	Mixed	To rebuild the provincial radiation therapy community to facilitate collaboration among centres, with the aim of decreasing variation in practice.	95% of participants reported that CoP projects were very relevant to them, and 50% reported changes in their practice due to the CoP. 90% reported growth in their professional network and 93% felt the CoP was worthwhile.
[42]	Med	2019	Bermejo-Caja et al.	Spain	12	Qualitative Research	Qualitative	To improve the attitude of primary care professionals to the empowerment of patients with chronic conditions	GPs found the vCoP useful as it could provide up to date resources that could be used at the point of care. Both professionals found that discussing experiences with others helped them consider alternative approaches and advance learning.
[43]	High	2019	Savoie et al.	Canada	77	Non-randomised experimental study	Quantitative	To improve pain practices for spinal cord injury patients	Adherence to pain best practices for SCI exceeded 70% for most outcomes, all of which were improvements on the retrospective cohort. This included improvements in developing inter-professional pain treatment plans from 12% to 74%, and documenting pain onset from 4.5% to 80%.
[44]	Low	2020	Rolls et al.	Australia	133	Qualitative Research	Mixed	To facilitate communication and knowledge sharing between the clinicians working at the 43 adults ICUs in New South Wales	Nurses contributed 68% of posts and physicians 27%. Knowledge supplied was either experiential (35%), explicit (17%), both (17%), know-how (20%), know-why (5%), or no-knowledge exchanged (6%). Three subject areas were identified: clinical practices (71%); equipment (23%); and clinical governance (6%). Six elements facilitated participation and knowledge exchange: discussion thread, sharing of artefacts, community, cordiality, maven work, and promotion of the community.
[45]	High	2020	Pariser et al.	Canada	616	Qualitative Research	Mixed	To provide streamlined access to specialist care and virtual-team based resources for primary care.	A CoP was successfully formed between primary care and specialist care. This also led to new initiatives being created that responded to primary care needs, such as facilitating real time access to radiology services. These initiatives led to a perceived reduction in ED visits by 40%.
[46]	Low	2020	McCurtin et al.	Ireland	15	Qualitative Research	Qualitative	To encourage clinician research engagement by linking them with researchers in higher educations institutions	Members of the CoP felt the priorities (in order) of the CoP should be: dissemination, education, enablers, networking, and advocacy. Actions proposed included the development of a research database, to act as advocates, as well as lobbying for clinical-research posts.
[47]	High	2021	Hahn-Golberg et al.	Canada	/	Non-randomised experimental study	Mixed	To implement the patient orientated discharge summary	High participation in the community of practice was associated with higher penetration. 64% of patents across the hospitals received a patient orientated discharge summary (PODS). PODS improved family involvement during discharge teaching (7% increase. p = 0.026).
[48]	Med	2021	Katzman et al.	USA	1530	Non-randomised experimental study	Mixed	To provide education for first responders on self-care techniques and stress resilience.	Overall stress levels did not decline, but participants felt more confident in using psychological first aid, managing others who needed mental health assistance, and taking time for self-care. They also had a significant reduction in how isolated they felt.
[49]	Med	2021	Dinesen et al.	Denmark	20	Non-randomised experimental study	Mixed	To improve rehabilitation of patients with AF	Patients found the program useful and felt more secure living with AF. Patients also displayed increased knowledge about AF at follow-up compared with baseline (p = 0.02).
[50]	High	2021	Keir et al.	Australia	3228	Non-randomised experimental study	Quantitative	To facilitate the spread of information regarding neonatal evidence based medicine	Since the registration of the hashtag, it has been used in 23939 tweets and 37259710 impressions were generated. The majority of users made one tweet using the hashtag (n = 1078), followed by two tweets (n = 411), and more than 10 tweets (n = 347). The online community contained the critical components of a community of practice.
[51]	Med	2022	Steinbock et al.	USA	90	Non-randomised experimental study	Quantitative	To increase viral suppression rates in populations disproportionately affected by HIV	The average viral suppression rates for the selected populations increased from 79.2% to 82.3%. The viral suppression gap between the selected disadvantaged groups and the rest of the served HIV population was reduced from 5.7% to 3.8%, a 33.5% reduction.
[52]	Low	2021	Gerritsen et al.	Netherlands	101	Qualitative Research	Qualitative	To support the implementation of the psychiatric intensive care approaches.	Key insights included the need to create an ambassador role for CoP participants, to organise concrete activities, be mindful of the multi-disciplinary composition, to foster shared responsibility, and to work on sustainability. The CoP was perceived to help support and further develop the HIC and FHIC approaches.
[53]	Low	2022	Montali et al.	Italy	16	Qualitative Research	Qualitative	To give breast cancer patients a space to talk about their experiences and receive peer support.	Analysis revealed five processes that breast cancer patients go through including: mirroring, monitoring, modelling, belonging, and distancing. The community contributed to the participants’ sense of empowerment.
[54]	Low	2022	Dames et al.	Canada	94	Non-randomised experimental study	Mixed	To deliver a 12 week ketamine-assisted therapy program	Pre post scores: PHQ-9 13 (moderate) to 7 (mild), PCL-5 47 (moderate) to 20 (mild), GAD-7 12 (moderate) to 6 (mild), B-IPF 42 (moderate) to 18 (mild). 91% of GAD and 79% of depression went into a milder category. 86% of PTSD screen negative and 92% of those with life work impairments had significant improvements.
[55]	Low	2022	Rushanan et al.	USA	13	Non-randomised experimental study	Mixed	To build the competence of occupational therapists treating patients with neurodegenerative diseases	The clinical competency assessment tool for occupational therapists treating patients with neurodegenerative diseases (CAT) for knowledge improved from 26.9 to 35.7 (p = 0.002), for beliefs improved from 28.7 to 35.2 (p = 0.001), and for actions improved from 25.2 to 31.9 (p = 0.002).
[56]	Med	2022	Sibbald et al.	Canada	17	Non-randomised experimental study	Mixed	To connect mid-career professionals from across Canada who are committed to improving healthcare police and practice	The program was successful in helping participants make connections (mean = 2.43). Participants reported the development of a sense of belonging (mean = 2.29) and facilitated knowledge exchange (mean = 2.43). At the time of this study, participants felt the program had minor impact on their work (mean = 3.5).

Developing skills was also a common reason for setting up a CoP with 8 studies in this theme. This included building research skills [29, 32] and developing self-care techniques [48]. There were also 7 studies whose aim was to share best-practice. This included the direct sharing of evidence-based practice [12, 50] as well as trying to decrease variation in practice over geographically spread out areas by providing clinicians in the same speciality a means of communication [11, 41, 44].

Sharing specialist knowledge was the aim of 6 studies. Of these, 4 were aimed at connecting primary care physicians with hospital-based specialists [14, 27, 39, 45] for example providing rural primary care physicians the knowledge to manage patients with chronic hepatitis C infection [14]. Another 3 studies brought clinicians together with researchers with the aim to stimulate research ideas and activity [8, 36, 46].

Other notable CoPs were set up with the specific aim to complete a specific task, such as develop a set of standards for genetic counselling in Europe [15], or to co-design a smartphone application with patients for schizophrenia care [33].

### Effectiveness of the CoPs

The effectiveness of the CoPs was measured in a variety of ways (Table 1). 30 studies were qualitative research, 20 studies used a non-randomised experimental design, and 1 study was a randomised controlled trial [25]. In terms of outcomes, qualitative outcomes were the most common measure used in 21 studies, a mix of both qualitative and quantitative outcomes were used in 20 studies, and solely quantitative outcomes were used in 9 studies. Only 11 of the studies with a quantitative element had the appropriate statistical methodology to report significance. All except 1 study [40] reported a positive significant effect when implementing a CoP. Outcomes varied across geographical location with North American countries such as Canada (91.7%) and USA (80%) having a higher percentage of studies with a quantitative element to their outcomes, in comparison to the UK (12.5%) or Australia (50%).

Of the 9 studies that showed a statistically significant effect, 5 showed improvements in hospital-based provision of services [12, 25, 35, 47, 49, 55]. These studies included implementing patient orientated discharge summaries leading to an 7% increase (p = 0.026) of family involvement during discharge [47], as well as another study improving rehabilitation services for patients with atrial fibrillation (AF) which demonstrated an increase in patients’ knowledge about AF (p = 0.02) [49]. 2 of the studies showed improvements in primary-care. Arora et al. showed how bringing primary care providers together with hospital specialists improved primary care knowledge about the management of hepatitis C infection (p<0.0001). Diaz-Chao et al. showed how bringing primary care physicians together with specialists led to fewer hospital referrals (p<0.05). Finally, 2 studies showed improvements in direct clinical outcomes. One study showed a reduction in central line infections by 50% (p<0.05) (9) and another showed an increase in HIV viral suppression rates from 79.2% to 82.3% (p<0.05) [51].

### Communication

Table 2 describes the methods of communication utilised by each of the communities of practice described in the 50 studies included in this review. Of the communities of practice 23 communicated virtually, 12 communicated face-to-face, and 13 used both face to face and virtual methods of communication. In two of the studies [26, 41], it is unclear whether the communication was virtual, face-to-face or both. 23 of the communities of practice held meetings for the members, 10 utilised workshops, 8 described seminars, and 1 described tutorials [42]. 25 studies communicated using web-based systems and blogs and 10 communicated via email. 18 of the studies described other methods of communication, which included video consultation [49], telephone-based catch-ups [14, 18] and case based presentations/discussions [14, 19, 23, 51]. The average year for face-to-face only communication was 2014.25 (SD 4.94) and 2015.78 (SD 5.56) for virtual only communication, which was not significantly different (p = 0.43).

**Table 2:** Methods of communication.

Ref	Face-to-Face	Virtual	Workshops	Seminars	Meeting of Members	Emails	Web Based Systems and Blogs	Other
[7]		Yes		Yes				
[8]		Yes				Yes		Personalised targeting of content based on interests
[9]	Yes		Yes		Yes			Presentation from the monthly members meeting posted on bulletin boards.
[10]	Yes				Yes			
[11]		Yes				Yes	Yes	
[12]	Yes	Yes			Yes		Yes	
[13]	Yes							Organically working together on the ward
[14]		Yes						Weekly 2hr telemedicine clinics
[15]	Yes	Yes			Yes	Yes	Yes	
[16]	Yes				Yes			
[17]		Yes					Yes	
[18]	Yes							Over the phone catch-ups
[19]	Yes	Yes						Case-conferences
[20]		Yes				Yes	Yes	Document and image repository
[21]	Yes				Yes			
[22]		Yes						
[23]		Yes		Yes				Case-based discussions
[24]		Yes			Yes			
[25]		Yes		Yes		Yes	Yes	
[26]					Yes			
[27]		Yes				Yes	Yes	Document and image repository
[28]		Yes					Yes	
[29]	Yes	Yes					Yes	
[30]	Yes	Yes		Yes	Yes		Yes	Journal club
[31]	Yes				Yes			
[32]	Yes	Yes	Yes				Yes	
[33]	Yes		Yes		Yes	Yes		
[34]	Yes	Yes	Yes				Yes	
[35]	Yes	Yes	Yes				Yes	
[36]	Yes	Yes			Yes		Yes	Speed dating, keynote speaker, and meeting feedback
[37]		Yes					Yes	
[38]	Yes				Yes			
[39]		Yes				Yes	Yes	
[40]	Yes	Yes		Yes			Yes	Critical care quality day
[41]								
[42]		Yes		Yes			Yes	
[43]	Yes		Yes		Yes			
[44]		Yes				Yes		
[45]	Yes	Yes	Yes		Yes	Yes	Yes	
[46]	Yes		Yes		Yes			
[47]		Yes			Yes		Yes	Mentorship
[48]		Yes		Yes	Yes			Weekly learning-listening sessions
[49]	Yes	Yes		Yes			Yes	
[50]		Yes					Yes	
[51]		Yes		Yes	Yes		Yes	Case presentations
[52]	Yes		Yes		Yes			
[53]		Yes					Yes	
[54]	Yes	Yes			Yes			
[55]		Yes			Yes		Yes	
[56]		Yes			Yes			

### Barriers and facilitators

Barriers to engagement were reported in 15 of the studies; examples are given in Table 3. The biggest barrier to engagement was time constraints, reported in nine of the studies. Lack of space to meet up [18], or to access the vCOP [42] was reported in two of the studies, and lack of funding [43] or resource constraints [12] as a barrier was reported in two studies. Difficulty accessing the COP platform via usual workstations [39] or operating systems [42] was listed as a barrier in two of the studies. A lack of understanding of the concept of the COP was reported as a barrier in one study [10]; two studies cited fear of judgement as barriers to engagement [11, 53]. One study noted that those who were encouraged to join the COP by peers had lower engagement than those who self-selected [29], whilst another found that lack of participation by peripheral members caused frustration among core members [21].

**Table 3:** Barriers to engagement.

Barrier	Example	Ref
Time constraints	The time commitment was the biggest barrier	[41]
Space constraints	Barriers included… a lack of space to meet up	[18]
Resource constraints	A lack of funding resulted in longer implementation times	[43]
Information Technology constraints	Not having the tool integrated into usual work stations… proved to be a barrier	[39]
Lack of understanding	Barriers included not understanding the CoP concept	[10]
Fear of judgement	Barriers… included… concern about how interesting a case is, and showing a gap in one’s knowledge	[11]
Mode of selection	Those who were encouraged to join the CoP by peers, rather than self-selecting, had lower engagement	[29]

Facilitators were reported in 24 of the studies; examples are given in Table 4. The most commonly highlighted facilitators were involvement of key members of the team. Primarily these were clinical, with studies citing strong clinical leadership [26, 39], support from health [16] or hospital [9] leadership, clinical champions [11, 24], experts [34, 38], and involving PCPs in the early stages of development [45]. Non-clinical roles were also highlighted, with two of the studies listed having a group facilitator important for engagement [21, 42], and one highlighting the importance of funding for an administrative coordinator [36]. One study found that a mentoring scheme helped to distribute expertise [36], whilst another found the opportunity for new members to learn through passive participation to be a facilitator [8]. Regular face to face meetings were listed by three of the studies as facilitators [36, 38, 49], with one study noting that using face-to-face and virtual activities supported different learning styles [35]. Use of social networks and ICT technologies in professional practice were found to be facilitators in one of the studies [36]. Alignment with existing practices, in particular with quality improvement methodology, was noted to be a facilitator in two of the studies [24, 29].

**Table 4:** Facilitators of engagement.

Facilitator	Example	Ref
Clinical leadership	Strong clinical leadership was the most important success factor	[26]
Hospital/health leadership support	Support of the CoP by health leaders was a major facilitator	[16]
Expert knowledge	Facilitators included access to a panel of experts	[34]
Group facilitators	The facilitator motivated members to contribute and filtered in relevant information	[42]
Clinical champions	Facilitators included the existence of a champion in the field	[11]
Administrative coordinator	The funding for the administrative co-ordinator has been a facilitator	[36]
Quality improvement methodology	Methodology that closely resembled quality improvement and allowed for quick wins kept the groups engaged	[29]

## Discussion

This systematic review has elucidated the aims and effectiveness of CoPs established in a healthcare setting. As described above, there were a variety of aims for the CoPs, with the majority relating to improving clinical outcomes and knowledge. Although encouraging to see the focus of these CoPs on clinically relevant issues, there were only 3 studies [9, 40, 51] where the outcome measurement was a patient related clinical outcome with the suitable statistical methodology to determine a significant effect.

Furthermore, only 1 study [25] had a randomised control trial (RCT) design and therefore the ability to establish causality. In this study, 122 veterans affairs (VA) hospitals were randomised to have enrolment into a new initiative facilitated either by a CoP or through usual means—the standard national announcements that all hospitals receive for new initiatives. The initiative was the national hospital to home (H2H) project, and uptake to the programme was the primary outcome measure. H2H aimed to help inpatients with heart disease transition back to their place of residence through measures such as early follow up and patient education to recognise early signs of deterioration. The CoP was an already existing entity that had been set up previously to connect VA hospitals to improve the quality of care for patients with heart disease. The primary means of communication of the CoP was via email and they also had bimonthly teleconferences. 54% of the hospitals randomised to CoP facilitated arm enrolled in the H2H initiative whereas only 10% of those not facilitated by the CoP enrolled (p < 0.001). This is clear evidence of the effectiveness of utilising CoPs, albeit indirectly, for changing clinical practice. However, the ultimate goal of the H2H was to reduce 30-day readmission rates by 20%, and this study did not measure and compare this, which would have provided a more clinically meaningful endpoint.

Although not formally described as CoPs, and therefore were not picked up in the systematic search of this review, there are other RCTs published in the literature that provide support for the effectiveness of CoPs directly on clinical outcomes. Described as peer-mentoring schemes or online communities, lifestyle interventions that bring patients together who share the same set of problems, such as poor glucose control or low activity levels, have been highly effective at motivating patients to alter their behaviour [57, 58]. Richardson et al. conducted an RCT that provided the intervention arm with means of communication with their fellow patients during an online intervention to increase physical activity. Those able to communicate with their fellow participants, through posting and reading messages on a web-based blog, had a significantly reduced attrition rate than those who had no means of communication (79% v 56%, p = 0.02).

As the most common outcome of the CoPs was a change in practice, it is clear that as well as being a knowledge management strategy, CoPs are also behaviour change interventions. The capability, opportunity, and motivation model of behaviour change (COM-B) is a systematic way of framing the different facets required for an individual to change their behaviour [59]. Capability is defined as the psychological and physical requirements to perform the task. Opportunity represents the physical and social factors outside of the individual that make the behaviour possible, and motivation is defined as both the reflective and automatic brain activity that energises and directs behaviour. Through the community and shared problem solving that CoPs offer, it is clear that they provide individuals with the psychological capability, social opportunity, and motivation they need for behaviour change through the learning resources and peer support available.

The main barrier to engagement was time constraints, which are to be expected in an overwhelmed healthcare environment that is busier than ever [60]. Funding constraints were also noted, which once again is not a surprise as healthcare spending as a percentage of GDP is at its lowest in a decade [61]. It is, however, encouraging to see that there were no barriers relating to a lack of digital skills, despite many individuals known to struggle [62]. With the digital revolution taking place in healthcare, strong digital skills in the workforce will be necessary to control spiralling costs. Such skills will be necessary for vCoPs to be taken up in a meaningful way across the healthcare ecosystem.

The main facilitator for engagement was strong leadership, including support from institutional leaders, which represents an alteration from the original vision for CoPs as self-organising entities with a lack of centralised leadership. This shows the specific healthcare related factors that many interventions face in the highly regulated and controlled environment. Future CoP endeavours should bear this in mind and make sure support and buy in is gained from the relevant clinical and administrative leaders. This will also help alleviate the main barrier to engagement by providing support or even specific protected time for the CoP.

CoPs differ from other knowledge management strategies such as work groups or knowledge networks. In work groups goals are pre-determined by a manager and members are usually assigned or selected by a leader. CoPs on the other hand goals are negotiated by members and membership is self-selecting, by identifying with the domain of knowledge of the CoP. Knowledge networks are at the other end of the spectrum to work groups and are an informal set of relationships which are primarily concerned with passing on knowledge, rather than the full range of knowledge management. In comparison, CoPs have a shared mission and desire in its members to work together to deepen their knowledge. CoPs also focus on the creation, storage, and utilisation of knowledge.

This review had a number of limitations. Only studies that directly had mention of a community of practice in the title, abstract, or full text were included. This meant that the diverse array of names used to refer to the concept of CoPs, such as situated learning, learning network, or even just community, were not included potentially excluding valuable studies. However, these phrases are used too ubiquitously in the field of healthcare, and as such, so broad a search was not feasible and so the search was focussed solely on the term community/ies of practice. Studies regarding medical education were also excluded, as has similarly been done in previous reviews [63], as these participants wouldn’t necessarily be involved in providing healthcare directly. However, these studies may still have provided insights into the barriers and facilitators of engagement with healthcare themed CoPs. This review also did not employ a snowballing technique to examine the full list of references in each included study to broaden the search methodology. It was also not technically possible to carry out a logistic regression looking for the factors that were associated with effective CoPs as only 1 study reported a negative result.

CoPs in healthcare are aimed at improving clinical outcomes and have been shown to be effective. There is still progress to be made and a need for further studies with more rigorous methodologies, such as RCTs, to provide further support of the causality of CoPs on outcomes. As healthcare systems continue through their digital transformation journeys and healthcare workers have to manage an ever-mounting amount of knowledge, vCoPs in particular offer a method for improving outcomes and sharing vital information across an ever more complex healthcare landscape.

## Supporting information

S1 Checklist PRISMA 2020 checklist. (DOCX)

## References

[1] Densen P. Challenges and opportunities facing medical education. Transactions of the American Clinical and Climatological Association. 2011;122:48. 21686208 PMC3116346

[2] Girard J, Girard J. Defining knowledge management: Toward an applied compendium. Online Journal of Applied Knowledge Management. 2015 Jan;3(1):1–20.

[3] Wenger E., McDermott R. A., & Snyder W. (2002). Cultivating communities of practice: A guide to managing knowledge. Harvard business press.

[4] Lesser EL, Storck J. Communities of practice and organizational performance. IBM systems journal. 2001;40(4):831–41.

[5] Polanyi M. The Tacit dimension, knowledge in organizations. Prusak L., Ed. 1966:135–46.

[6] Gabbay J, le May A. Mindlines: making sense of evidence in practice. British Journal of General Practice. 2016 Aug 1;66(649):402–3. doi: 10.3399/bjgp16X686221 27481961 PMC4979949

[7] Gagliardi A, Smith A, Goel V, DePetrillo D. Feasibility study of multidisciplinary oncology rounds by videoconference for surgeons in remote locales. BMC medical informatics and decision making. 2003 Dec;3:1–7. 12816548 10.1186/1472-6947-3-7 PMC165596

[8] Russell J, Greenhalgh T, Boynton P, Rigby M. Soft networks for bridging the gap between research and practice: illuminative evaluation of CHAIN. Bmj. 2004 May 13;328(7449):1174. doi: 10.1136/bmj.328.7449.1174 15142924 PMC411097

[9] Render ML, Brungs S, Kotagal U, Nicholson M, Burns P, Ellis D et al. Evidence-based practice to reduce central line infections. The Joint Commission Journal on Quality and Patient Safety. 2006 May 1;32(5):253–60. doi: 10.1016/s1553-7250(06)32033-8 16761789

[10] White D, Suter E, Parboosingh IJ, Taylor E. Communities of practice: Creating opportunities to enhance quality of care and safe practices. Healthcare Quarterly. 2008 Mar 15;11(3):80–4. doi: 10.12927/hcq.2008.19654 18382166

[11] Falkman G, Gustafsson M, Jontell M, Torgersson O. SOMWeb: a semantic web-based system for supporting collaboration of distributed medical communities of practice. Journal of medical Internet research. 2008 Aug 26;10(3):e1059. doi: 10.2196/jmir.1059 18725355 PMC2626431

[12] Tolson D, Booth J, Lowndes A. Achieving evidence‐based nursing practice: impact of the Caledonian Development Model. Journal of nursing management. 2008 Sep;16(6):682–91. doi: 10.1111/j.1365-2834.2008.00889.x 18808462

[13] Griffiths P. A community of practice: the nurses’ role on a medical assessment unit. Journal of Clinical Nursing. 2011 Jan;20(1‐2):247–54. doi: 10.1111/j.1365-2702.2009.03135.x 20626529

[14] Arora S, Kalishman S, Thornton K, Dion D, Murata G, Deming P et al. Expanding access to hepatitis C virus treatment—Extension for Community Healthcare Outcomes (ECHO) project: disruptive innovation in specialty care. Hepatology. 2010 Sep;52(3):1124–33. doi: 10.1002/hep.23802 20607688 PMC3795614

[15] Skirton H, Patch C, Voelckel MA. Using a community of practice to develop standards of practice and education for genetic counsellors in Europe. Journal of Community Genetics. 2010 Dec;1:169–73. doi: 10.1007/s12687-010-0024-y 22460299 PMC3186000

[16] Burgess J, Sawchenko L. Community of practice: a nurse practitioner collaborative model. Nursing Leadership (Toronto, Ont.). 2011 Jun 1;24(2):99–112. doi: 10.12927/cjnl.2011.22468 21730772

[17] Massett HA, Parreco LK, Padberg RM, Richmond ES, Rienzo ME, Leonard CE et al. AccrualNet: Addressing low accrual via a knowledge-based, community of practice platform. Journal of oncology practice. 2011 Nov;7(6):e32–9. doi: 10.1200/JOP.2011.000272 22379429 PMC3219473

[18] Adams M, Robert G, Maben J. ‘Catching up’: The significance of occupational communities for the delivery of high quality home care by community nurses. Health:. 2013 Jul;17(4):422–38. doi: 10.1177/1363459312460703 23079580

[19] Fung-Kee-Fung M, Boushey RP, Watters J, Morash R, Smylie J, Morash C et al. Piloting a regional collaborative in cancer surgery using a “community of practice” model. Current oncology. 2014 Feb;21(1):27–34. doi: 10.3747/co.21.1663 24523602 PMC3921028

[20] Díaz-Chao Á, Torrent-Sellens J, Lacasta-Tintorer D, Saigí-Rubió F. Improving integrated care: modelling the performance of an online community of practice. International journal of integrated care. 2014 Jan;14. doi: 10.5334/ijic.1200 24648835 PMC3952812

[21] Bindels J, Cox K, Widdershoven G, van Schayck CP, Abma TA. Stimulating program implementation via a Community of Practice: a responsive evaluation of care programs for frail older people in the Netherlands. Evaluation and Program planning. 2014 Oct 1;46:115–21. doi: 10.1016/j.evalprogplan.2014.06.001 24974372

[22] Carolan I, Smith T, Hall A, Swallow VM. Emerging communities of child-healthcare practice in the management of long-term conditions such as chronic kidney disease: qualitative study of parents’ accounts. BMC Health Services Research. 2014 Dec;14:1–9. 25001236 10.1186/1472-6963-14-292 PMC4107554

[23] Meins AR, Doorenbos AZ, Eaton L, Gordon D, Theodore B, Tauben D. TelePain: A community of practice for pain management. Journal of pain & relief. 2015 Mar 3;4(2). doi: 10.4172/2167-0846.1000177 25964869 PMC4425941

[24] Shaikh U, Romano P, Paterniti DA. Organizing for quality improvement in health care: an example from childhood obesity prevention. Quality management in health care. 2015 Jul 1;24(3):121–8. doi: 10.1097/QMH.0000000000000066 26115059

[25] Heidenreich PA, Sahay A, Mittman BS, Oliva N, Gholami P, Rumsfeld JS et al. Facilitation of a multihospital community of practice to increase enrollment in the hospital to home national quality improvement initiative. The Joint Commission Journal on Quality and Patient Safety. 2015 Aug 1;41(8):361–9. doi: 10.1016/s1553-7250(15)41047-5 26215525

[26] Jefford M, Kinnane N, Howell P, Nolte L, Galetakis S, Bruce Mann G et al. Implementing novel models of posttreatment care for cancer survivors: enablers, challenges and recommendations. Asia‐Pacific Journal of Clinical Oncology. 2015 Dec;11(4):319–27. doi: 10.1111/ajco.12406 26245952

[27] Lacasta Tintorer D, Flayeh Beneyto S, Manresa JM, Torán-Monserrat P, Jiménez-Zarco A, Torrent-Sellens J et al. Understanding the discriminant factors that influence the adoption and use of clinical communities of practice: the ECOPIH case. BMC Health Services Research. 2015 Jun;15(1):1–0. doi: 10.1186/s12913-015-1036-4 26358037 PMC4566431

[28] Dong C, Cheema M, Samarasekera D, Rajaratnam V. Using LinkedIn for continuing community of practice among hand surgeons worldwide. Journal of Continuing Education in the Health Professions. 2015 Jul;35(3):185–91. doi: 10.1002/chp.21300 26378424

[29] Gullick JG, West SH. Building research capacity and productivity among advanced practice nurses: an evaluation of the Community of Practice model. Journal of advanced nursing. 2016 Mar;72(3):605–19. doi: 10.1111/jan.12850 26537181

[30] McCreesh K, Larkin L, Lewis J. Shouldering the burden of evidence-based practice: the experiences of physiotherapists partaking in a Community of Practice. Rehabilitation Research and Practice. 2016 Jan 24;2016. 10.1155/2016/9051378 PMC4745822 26904293

[31] Wallis SK, Jehan K, Woodhead M, Cleary P, Dee K, Farrow S et al. Health professionals’ experiences of tuberculosis cohort audit in the North West of England: a qualitative study. BMJ open. 2016 Mar 1;6(3):e010536. doi: 10.1136/bmjopen-2015-010536 26983949 PMC4800141

[32] Becerril-Montekio V, Alcalde-Rabanal J, Darney BG, Orozco-Nuñez E. Using systematized tacit knowledge to prioritize implementation challenges in existing maternal health programs: implications for the post MDG era. Health policy and planning. 2016 Oct 1;31(8):1031–8. doi: 10.1093/heapol/czw033 27060787 PMC5013782

[33] Terp M, Laursen BS, Jørgensen R, Mainz J, Bjørnes CD. A room for design: Through participatory design young adults with schizophrenia become strong collaborators. International journal of mental health nursing. 2016 Dec;25(6):496–506. doi: 10.1111/inm.12231 27293176

[34] Francis-Coad J, Etherton-Beer C, Bulsara C, Nobre D, Hill AM. Using a community of practice to evaluate falls prevention activity in a residential aged care organisation: a clinical audit. Australian health review. 2016 Mar 17;41(1):13–8. 10.1071/AH15189 26982888

[35] Camden C, Rivard LM, Hurtubise K, Héguy L, Berbari J. Can a community of practice improve physical therapists’ self-perceived practice in developmental coordination disorder?. Physical Therapy. 2017 Jul 1;97(7):746–55. doi: 10.1093/ptj/pzx041 28444245

[36] Cheng A, Auerbach M, Calhoun A, Mackinnon R, Chang TP, Nadkarni V et al. Building a community of practice for researchers: the international network for simulation-based pediatric innovation, research and education. Simulation in Healthcare. 2018 Jun 1;13(3S):S28–34. 29117090 10.1097/SIH.0000000000000269

[37] Wieringa S, Engebretsen E, Heggen K, Greenhalgh T. How knowledge is constructed and exchanged in virtual communities of physicians: qualitative study of mindlines online. Journal of medical Internet research. 2018 Feb 2;20(2):e34. doi: 10.2196/jmir.8325 29396385 PMC5882224

[38] Fingrut W, Beck LA, Lo D. Building an oncology community of practice to improve cancer care. Current Oncology. 2018 Dec;25(6):371–7. doi: 10.3747/co.25.4087 30607111 PMC6291277

[39] Lacasta Tintorer D, Manresa Domínguez JM, Pujol-Rivera E, Flayeh Beneyto S, Mundet Tuduri X, Saigí-Rubió F. Keys to success of a community of clinical practice in primary care: a qualitative evaluation of the ECOPIH project. BMC family practice. 2018 Dec;19:1–3. 29743030 10.1186/s12875-018-0739-0 PMC5944103

[40] Dodek P, McKeown S, Young E, Dhingra V. Development of a provincial initiative to improve glucose control in critically ill patients. International Journal for Quality in Health Care. 2019 Feb 1;31(1):49–56. doi: 10.1093/intqhc/mzy101 29757412

[41] Glicksman R, Ang M, Murray E, Simniceanu C, Lockhart E, Gilbert J et al. Improving quality of radiation therapy care across Ontario using a community-of-practice approach. Practical radiation oncology. 2019 Mar 1;9(2):e242–8. doi: 10.1016/j.prro.2018.10.017 30447404

[42] Bermejo-Caja CJ, Koatz D, Orrego C, Perestelo-Pérez L, González-González AI, Ballester M et al. Acceptability and feasibility of a virtual community of practice to primary care professionals regarding patient empowerment: a qualitative pilot study. BMC Health Services Research. 2019 Dec;19(1):1–0. 31221215 10.1186/s12913-019-4185-z PMC6587268

[43] Savoie J, McCullum S, Wolfe DL, Slayter J, O’Connell C. Implementation of pain best practices as part of the spinal cord injury knowledge mobilization network. The Journal of Spinal Cord Medicine. 2019 Sep 30;42(sup1):226–32. doi: 10.1080/10790268.2019.1654191 31573455 PMC6781186

[44] Rolls KD, Hansen MM, Jackson D, Elliott D. Intensive care nurses on social media: An exploration of knowledge exchange on an intensive care virtual community of practice. Journal of clinical nursing. 2020 Apr;29(7–8):1381–97. doi: 10.1111/jocn.15143 31856353 PMC7328784

[45] Pariser P, Gush H, Ivers N, Pomedli S. Building a Primary Care Community of Practice: SCOPE as a Platform for Care Integration and System Transformation. Healthcare Quarterly (Toronto, Ont.). 2020 Jul 1;23(2):44–9. doi: 10.12927/hcq.2020.26275 32762820

[46] McCurtin A O’Connor A. Building a collaborative research community of practice and supporting research engagement in speech-language pathology: identification of stakeholder priorities. JBI Evidence Implementation. 2020 Dec 1;18(4):368–75. doi: 10.1097/XEB.0000000000000229 33570320

[47] Hahn-Goldberg S, Huynh T, Chaput A, Krahn M, Rac V, Tomlinson G et al. Implementation, spread and impact of the Patient Oriented Discharge Summary (PODS) across Ontario hospitals: a mixed methods evaluation. BMC Health Services Research. 2021 Dec;21(1):1–4. 33865385 10.1186/s12913-021-06374-8 PMC8052788

[48] Katzman JG, Tomedi LE, Everly G, Greenwood-Ericksen M, Romero E, Rosenbaum N et al. First responder resiliency echo: innovative telementoring during the covid-19 pandemic. International journal of environmental research and public health. 2021 May 4;18(9):4900. doi: 10.3390/ijerph18094900 34064501 PMC8124662

[49] Dinesen B, Dam Gade J, Skov Schacksen C, Spindler H, Eie Albertsen A, Dittmann L et al. The Danish Future Patient Telerehabilitation Program for Patients With Atrial Fibrillation: Design and Pilot Study in Collaboration With Patients and Their Spouses. JMIR cardio. 2021 Jul 19;5(2):e27321. doi: 10.2196/27321 34279239 PMC8329756

[50] Keir A, Bamat N, Hennebry B, King B, Patel R, Wright C et al. Building a community of practice through social media using the hashtag# neoEBM. Plos one. 2021 May 28;16(5):e0252472. doi: 10.1371/journal.pone.0252472 34048469 PMC8162580

[51] Steinbock CM, Chung R, Lee JE, Leung SY, Kolesar C, Tesoriero J. Reducing Disparities: A Virtual Quality Improvement Collaborative Resulted in Better Health Outcomes for 4 Target Populations Disproportionately Affected by HIV. Journal of Public Health Management and Practice. 2022 Mar 1;28(2):162–9. doi: 10.1097/PHH.0000000000001360 33938485

[52] Gerritsen S, Van Melle AL, Zomer LJ, Widdershoven GA, Voskes Y. Communities of Practice in Acute and Forensic Psychiatry: Lessons Learned and Perceived Effects. Psychiatric Quarterly. 2021 Dec;92(4):1581–94. doi: 10.1007/s11126-021-09923-w 34109492 PMC8531102

[53] Montali L, Zulato E, Frigerio A, Frangi E, Camussi E. Mirroring, monitoring, modelling, belonging, and distancing: Psychosocial processes in an online support group of breast cancer patients. Journal of Community Psychology. 2022 Mar;50(2):992–1007. doi: 10.1002/jcop.22696 34428308 PMC9290070

[54] Dames S, Kryskow P, Watler C. A cohort-based case report: The impact of ketamine-assisted therapy embedded in a community of practice framework for healthcare providers with PTSD and depression. Frontiers in Psychiatry. 2022:2391. 10.3389/fpsyt.2022.962882 PMC9344338 35928772

[55] Rushanan SG, Nilsen DM, Grajo L, Caroll K. Assessing and Building Clinical Competence in Occupational Therapists Treating Patients with Neurodegenerative Disease: A Community of Practice Study. Occupational Therapy In Health Care. 2022 Aug 3:1–21. doi: 10.1080/07380577.2022.2105470 35943279

[56] Sibbald SL, Burnet ML, Callery B, Mitchell JI. Building a virtual community of practice: experience from the Canadian foundation for healthcare improvement’s policy circle. Health Research Policy and Systems. 2022 Sep 1;20(1):95. doi: 10.1186/s12961-022-00897-0 36050686 PMC9434556

[57] Long JA, Jahnle EC, Richardson DM, Loewenstein G, Volpp KG. Peer mentoring and financial incentives to improve glucose control in African American veterans: a randomized trial. Annals of internal medicine. 2012 Mar 20;156(6):416–24. doi: 10.7326/0003-4819-156-6-201203200-00004 22431674 PMC3475415

[58] Richardson CR, Buis LR, Janney AW, Goodrich DE, Sen A, Hess ML et al. An online community improves adherence in an internet-mediated walking program. Part 1: results of a randomized controlled trial. Journal of medical Internet research. 2010 Dec 17;12(4):e1338. 10.2196/jmir.1338 PMC3056526 21169160

[59] Michie S, Van Stralen MM, West R. The behaviour change wheel: a new method for characterising and designing behaviour change interventions. Implementation science. 2011 Dec;6(1):1–2. doi: 10.1186/1748-5908-6-42 21513547 PMC3096582

[60] https://www.england.nhs.uk/2021/12/busiest-november-ever-as-new-figures-show-pressures-on-nhs-staff/

[61] https://www.health.org.uk/news-and-comment/charts-and-infographics/health-spending-as-a-share-of-gdp-remains-at-lowest-level-in

[62] https://www.lloydsbank.com/banking-with-us/whats-happening/consumer-digital-index/essential-digital-skills.html

[63] Ranmuthugala G, Plumb JJ, Cunningham FC, Georgiou A, Westbrook JI, Braithwaite J. How and why are communities of practice established in the healthcare sector? A systematic review of the literature. BMC health services research. 2011 Dec;11(1):1–6. 21999305 10.1186/1472-6963-11-273 PMC3219728

