# Supplementary material for: The aims and effectiveness of communities of practice in healthcare: A systematic review
Source: PLoS One. 2023 Oct 10;18(10):e0292343. doi: 10.1371/journal.pone.0292343 (PMC10564133; doi:10.1371/journal.pone.0292343)
Supplement: Attachment — Submitted filename: Response to Reviewer.docx [file pone.0292343.s002.docx]

26^th^ August 2023

**Responses to editor:**

PONE-D-23-17541

The Aims and Effectiveness of Communities of Practice in Healthcare: A Systematic Review

Dear Dr Edward Adekola Oladele,

Thank you very much for the reviewer’s comments and for inviting us to submit a revised manuscript. Please find our responses below. In the attached document, our responses to the questions are in blue, whilst changes to the text are given in green. This process has helped to significantly improve the manuscript. We look forward to hearing back from you.

Yours Sincerely,

Alexander Noar

Journal Requirements:

1. Please ensure that your manuscript meets PLOS ONE's style requirements, including those for file naming. The PLOS ONE style templates can be found at

https://journals.plos.org/plosone/s/file?id=wjVg/PLOSOne_formatting_sample_main_body.pdf and

<https://journals.plos.org/plosone/s/file?id=ba62/PLOSOne_formatting_sample_title_authors_affiliations.pdf>

Thank you for bringing our attention to this. The manuscript has been amended in line with these guidelines.

2. Thank you for stating the following in the Competing Interests section:

"Usman Jaffer is founder and CEO of health-shared.com which is a platform that hosts

communities of practice for a number of healthcare related issues."

Please confirm that this does not alter your adherence to all PLOS ONE policies on sharing data and materials, by including the following statement: "This does not alter our adherence to PLOS ONE policies on sharing data and materials.” (as detailed online in our guide for authors http://journals.plos.org/plosone/s/competing-interests). If there are restrictions on sharing of data and/or materials, please state these. Please note that we cannot proceed with consideration of your article until this information has been declared.

Please include your updated Competing Interests statement in your cover letter; we will change the online submission form on your behalf.

Thank you for bringing our attention to this. The manuscript and cover letter have been amended to include this statement.

3. We note that Figure 2 in your submission contain copyrighted images. All PLOS content is published under the Creative Commons Attribution License (CC BY 4.0), which means that the manuscript, images, and Supporting Information files will be freely available online, and any third party is permitted to access, download, copy, distribute, and use these materials in any way, even commercially, with proper attribution. For more information, see our copyright guidelines: <http://journals.plos.org/plosone/s/licenses-and-copyright>.

Thank you for bringing our attention to this. We were unfortunately not able to obtain the copyright permission for the images in Figure 2 so we have removed it from the manuscript.

4. Please include a new copy of Table 1 and 2 in your manuscript; the current table is difficult to read. Please follow the link for more information: <https://blogs.plos.org/plos/2019/06/looking-good-tips-for-creating-your-plos-figures-graphics/>

Thank you for bringing our attention to this. New editable copies of Table 1 and 2 have been included in the manuscript in line with PLOS guidelines.

5. Please review your reference list to ensure that it is complete and correct. If you have cited papers that have been retracted, please include the rationale for doing so in the manuscript text, or remove these references and replace them with relevant current references. Any changes to the reference list should be mentioned in the rebuttal letter that accompanies your revised manuscript. If you need to cite a retracted article, indicate the article’s retracted status in the References list and also include a citation and full reference for the retraction notice.

Thank you for bringing our attention to this. References have been checked and are in line with PLOS guidelines.

Response to Reviewer #1

Materials and Methods

The search approach for primary research studies was quite broad from 1950 to 2022 and this approach was appropriate for the subject matter. However, it would be valuable to include detailed search strategies undertaken to include more geographies, and any differences observed in CoPs outcomes. It would also have been useful to present the framework for the review in more detail at this sage

Thank you for this comment. There was no restriction on geography for the search. A description of differences in observed CoP outcomes between countries has also been added to the manuscript

There was no restriction on geographical location

Outcomes varied across geographical location with North American countries such as Canada (91.7%) and USA (80%) having a higher percentage of studies with a quantitative element to their outcomes, in comparison to the UK (12.5%) or Australia (50%).

Discussion

The discussion section expanded to provide a deeper analysis of the results and the authors clearly highlighted the limitations of their search strategy and the implications on “excluding valuable studies”. The reader may appreciate a brief comparison of CoPs with systematic of other knowledge sharing approaches relevant to the healthcare sector.

Thank you for this comment. The discussion has been updated to include a comparison of CoPs to other knowledge management groups.

CoPs differ from other knowledge management strategies such as work groups or knowledge networks. In work groups goals are pre-determined by a manager and members are usually assigned or selected by a leader. CoPs on the other hand goals are negotiated by members and membership is self-selecting, by identifying with the domain of knowledge of the CoP. Knowledge networks are at the other end of the spectrum to work groups and are an informal set of relationships which are primarily concerned with passing on knowledge, rather than the full range of knowledge management. In comparison, CoPs have a shared mission and desire in its members to work together to deepen their knowledge. CoPs also focus on the creation, storage, and utilisation of knowledge.

Minor Comments

1. Language and Grammar:

Abstract: Spelling error noted

Thank you for this comment. The spelling error in the abstract has now been corrected.

have

Response to Reviewer #2

Abstract: The abstract is generally well written. There is a typo “hage” instead of “have”. Despite not being unstructured, the abstract does not clearly present the results. The last paragraph also seems to combine some statements that are part of background with a conclusion and implications or recommendations. For clarity, the authors could clearly articulate the findings, a conclusion, and some recommendations while still following the journals non-structured abstract policy.

Thank you for this comment. The abstract has been updated in line with your recommendations.

Methods: Concise and easy to follow. Was there any limit to the geographic scope of the review. If so please clarify especially as results were presented by country where the research was conducted or the author’s home county.

Thank you for this comment. There was no restriction on geography for the search. The manuscript has been updated to reflect this.

There was no restriction on geographical location

Results: Well arranged and easy to follow. Table 1 cannot be read and could be added as supplemental material. All other tables are clear and well labeled

Thank you for this comment. Table 1 has been converted into an easier to read format.
